# To the Dental Profession

**Published:** 1857

**Authors:** 


### TO THE DENTAL PROFESSION.

In appearing before you at this time, I must apologize for the lateness and irregularity of this issue, by informing you of the reasons of delay. The first, was want of time; my whole time being taken up in the business department, I could not find leisure to publish; besides, there were less calls for the "Reporter" to come out at the regular time than usual, the "Register" and "News Letter" both having been got out ahead of time, with a full report of the great "Dental Convention" at Boston—there was nothing of importance to be said, and, therefore, I said *nothing*.

To those interested, and I believe the entire Dental profession are to some extent, I would now state that I have labored hard for a year and a half to rid myself of every species of merchandise, except such as is connected with the fitting out of Dentists or the practice of the Dental profession. I have been told that it is all folly to depend upon the Dentists in the West to support a Depot—that many of them will send East and pay *more*, and that others will spend hours and days in constructing some instrument or apparatus—which they could buy for what they pay for the materials—in short, that a Dental Depot could be kept only as an auxiliary or appendage to some other business.

But I have always had a more exalted opinion of the business and the profession, and think the real cause of their sending elsewhere was because they could not have their wants supplied. At all events, I have resolved to make the *trial*, and see whether the profession will or will not appreciate the effort. My object shall be to keep a full assortment of *everything* that the profession can be likely to want, and "nothing else"! to have my time, attention and capital all *concentrated* in the one business, and to make No. 38 West Fourth, THE DENTAL DEPOT, not only of the West, but of the whole country.

The location is in the center of business—a large room, fine light and air, and everything arranged for the especial accommodation of the profession. I have an abiding faith in their discrimination and appreciation; at least, I will not be convinced of the unfeasibility of the plan, unless I am driven to it by want of encouragement.

### Teeth in Sets---Exchanging, etc.

It is our effort and desire to keep on hand, at all times, a *perfect* assortment of Teeth in full sets, sets of 14, sets of 6, 4, 2, and promiscuous.

In order to do this, we must have the coöperation of the profession.

We sometimes have Dentists come into our establishment, who would, for present convenience, take a single tooth from a full set, and leave us 27 promiscuous teeth; they stand in their own light, they do not take into consideration that, perhaps, the next day they may want the very set they have broken.

We sometimes send teeth to the country, and have them returned, with one or two teeth taken from a full set, thus leaving us with a stock of teeth depreciated far more than the entire value of those they have purchased. It is not because they wish to do us harm, but because they do not reflect. If such a disposition were indulged, it would be impossible to keep up a good assortment, no matter what amount we would keep on hand. If we kept a million of teeth, with such practice they would soon all be "promiscuous."

We are perfectly willing and always ready to *exchange* all and every tooth we sell, over and over, again and again, until the parties are satisfied, and the indications fulfilled; but whatever teeth are exchanged, we expect to be returned in reasonable time and in the same condition as we send them, viz: full sets in full sets, sets of 14 in sets of 14, sets of 6 in sets of 6, etc.

If viewed in the proper light, I think every Dentist in the country will agree with me in this, because it is the only way in which a perfect stock and assortment can be kept up, and they accommodated with exactly what is desired in the most important cases, viz: full sets and those approximating.

For those who wish to keep a full stock on hand, and purchase by the quantity, I will make as liberal a discount as any one in the United States, for the same manufactures of teeth.

### Gold.

The financial embarrassments which have swept over the whole country have so depreciated the market value of a large proportion of the currency, and in a like ratio increased the value of or premium on gold coin, that we have found it necessary to *advance* the price of *gold* and *silver* plate and solder. 18 carat gold plate, which we have been selling at 90 cts., we have now advanced to $1.00 per dwt., and others in the same propo rtion.

I trust that this state of things will be of short duration ; but while it continues, *gold* and all the *precious metals* will vary in price, subject to the *fluctuations* of the market.

Manufacturers of *Gold Foil* are also talking seriously of advancing the price of Foil, and indeed, if this state of things continues, they will be obliged to do so. If they advance, we must necessarily do so.

---

### Still Later.

Since writing our article on Gold, the prices have been advanced by the manufacturers of Gold Foil from $28 to $30 per oz.; therefore, Gold Foil sold heretofore at $28, will hereafter, until further notice, be $30. Chas. Abbey & Sons', and A. J. Watts & Co's remains at $32.

We are compelled to acknowlege these are awful times in money matters, but have an abiding confidence that it will not last long, and is all for the *best* in the *end!*

---

### Stand from Under.

We give below an extract from one of our city Counterfeit Detectors, and one of which the community should be proud, and show a proper appreciation for the manly, independent, impartial manner in which it is conducted, the energy and promptness with which the proprietors place before their readers valuable and reliable information :

STAND FROM UNDER!

The Bank of Pennsylvania, Philadelphia, one of the oldest and largest institutions in the United States, with liabilities amounting in the aggregate to millions of dollars, suspended payment on the 25th ult. It is the depositary of the State funds, and numerous Banks throughout the Western and Southern country have accounts there. The Girard Bank, another gigantic concern, suspended the same day, and all the other Philadelphia Banks resolved to suspend specie payments on the 26th. The country Banks must follow. This looks more like a general Bank suspension and commercial bankruptcy than any thing which had previously occurred. Again we say, "Stand from under!" everywhere. The financial volcano is in motion; look out for the ROCKS!

### OUR QUOTATIONS

Have been thoroughly altered to what we expected would be the general rates for a time; but the precarious state of affairs which continues, renders even the increased rates unreliable beyond the hour. Our quotations for New York are altogether useless at present; John Thompson himself can not fix rates there even for a day. In Philadelphia and Pittsburgh, the bulk of Pennsylvania money is at a discount of from 2 to 20 per cent., notwithstanding a law which requires the Interior Banks, under a severe penalty, to keep their notes at par in those cities. Illinois and Wisconsin is down to 20 and 30 per cent. in Philadelphia; Ohio, 5. For the present, our readers must depend, as we do, on the varying advices of the hour. We shall continue to issue bulletins almost daily, which may be had without charge, on application at our office.—*Paddock's Bank Mirror.*

---

### Gold! Gold!! Gold!!! Gold!!!!

### AND ALL THE PRECIOUS METALS,

### HAVE ADVANCED IN PRICE.

In consequence of the scarcity and high prices charged for gold and silver coin, all articles manufactured from the precious metals, have necessarily advanced.

| | |
|---|---|
| Gold plate, 18 carats, - - - | $1.00 per dwt. |
| Gold solder, 16 " - - - | 90 " |
| " " 14 " - - - | 80 " |

Other qualities in proportion.

| | |
|---|---|
| Silver plate and wire, - - - | $1.75 per oz. |
| " solder - - - - | 1.50 " |

These are *present rates;* we trust, however, that this state of things will be of short duration, when the old prices will, of course, be resumed.

Nearly all the manufacturers of Gold Foil have advanced $2 on the ounce, and if this continues, others will be compelled to do the same. We, of course, have no alternative but to *do likewise.*

### Crystal Gold Foil.

This Foil is manufactured by Messrs. A. J. Watts & Co., of Utica, N. Y., from their prepared or Crystal Gold—hence its name. Its peculiar claims are purity, mellowness, plasticity and adhesiveness to a degree hitherto only partially and accidentally attainable, but which they undertake to secure with uniformity. They warrant this Foil to be ABSOLUTELY PURE GOLD. Those who are in the habit of manipulating Crystal Gold, will need no suggestions as to the mode of using this Foil. To all others it is only needful to say, that it should be worked with FIRST CLASS CRYSTAL GOLD PLUGGERS, or with ordinary Foil pluggers, so altered as to have serrated and finely cut points, and that it is susceptible of much the same as Crystal Gold in all modes of using Foil, except, possibly, in the "cylindrical" mode of filling, for which it may prove too adhesive. To that class of Dentists who desire Gold Foil of perfect *purity*, great adhesive or welding properties, and a constantly uniform character, this Foil is offered as the desideratum. Price $32 per ounce.

Sold wholesale and retail, by JNO. T. TOLAND,
38 *West Fourth st., Cincinnati, O.*

---

### Chas. Abbey & Sons' Gold Foil.

This old established and standard Foil needs no commendation from us. Nearly every Dentist in the whole country has tried it and used it, and many who had strayed away after other idols have with heartfelt penitence returned to their "first love."

A full supply of No. 4 and No. 6 is always on hand, and any other numbers will be furnished to order. Price $32 per ounce.

Sold wholesale and retail, by JNO. T. TOLAND,
38 *West Fourth st., Cincinnati, O.*

---

### Jas. Leslie's Gold Foil.

We would call especial attention to the fine Gold Foil manufactured by our fellow-citizen, Mr. Jas. Leslie. It will be found to possess pliability, toughness, and purity, the essential qualities to make a solid filling. It is liked by all who have used it, and many prefer it to all others. Mr. Leslie refers to

DR. JAMES TAYLOR,
" WM. M. HUNTER,
" P. KNOWLTON.

We are prepared to furnish the profession this Foil in any desired quantity, at the manufacturer's prices. The price has been for several years $28 per ounce; but owing to the present *scarcity* and *high* price of *Gold coin*, it has been found necessary to advance the price. For the present, and until financial matters settle down to the old standard, the price is $30 per ounce.

JNO. T. TOLAND,
38 *West Fourth st., Cincinnati.*

---

### J. B. Dunlevy's Gold Foil

Is an article which we have sold for several years, and we are pleased to say it has given universal satisfaction wherever it has been used. It possesses all the good qualities of the best Foils in the market, and is free from the objections made against some brands.

There is no Foil of which we have had better opportunity to know, and none in which we have more confidence.

Mr. Dunlevy is a highly scientific man and a perfect gentleman—and, like his Foil, improves on acquaintance—the more you have to do with them, the better you will like them.

A good supply always on hand, at the Cincinnati Dental Depot, 38 West Fourth street, where it is sold wholesale and retail, by JNO. T. TOLAND.

---

### Morgan's Gold Foil.

This article of *Fine Gold Foil* we have been selling something over a year. Our attention was first directed to it by a Dentist in this city, doing a very extensive practice, who had at various times tried every manufacture of Foil in the market, and finally settled down on this as the "ne plus ultra" of perfection. We have sold large quantities of it within the last year, and have found it almost universally admired.

We would bespeak for it a fair trial by all who are not already familiar with its good qualities. Present price $30 per ounce.

Sold wholesale and retail by JNO. T. TOLAND,
38 *West Fourth st., Cincinnati.*

### Porcelain Teeth.

I have on hand and am receiving a large stock and splendid assortment of teeth, from the following celebrated manufacturers:

Jones, White & McCurdy,
N. Y. Teeth Manufacturing Co.,
Orum & Armstrong,
J. Klein.

My stock comprises the *finest selections* of all the best manufacturers, which I am prepared to sell at the following *very low prices:*

| | | |
|---|---|---|
| Gum Front Teeth, each, | - - - | 20 cents. |
| Gum Mo. and Bi. Teeth, each, | - - | 20 " |
| Plain Front do. do. | - - | 10 " |
| do. M. and B. do. do. | - - | 10 " |
| Continuous Gum Teeth, do. | - - | 10 " |
| Teeth for Gutta Percha work, each, | - | 10 " |
| Pivot Teeth, | - - - - | 8 " |

I give a discount of 3 per cent. on bills of $30 worth of teeth, 5 per cent. on bills of $50, and 10 per cent. on $100.

☞ *Dealers* will be supplied on liberal terms.

☞ Teeth carefully selected to casts, or according to samples and directions, without extra charge.

---

### Block Work.

Executed in the most beautiful and artistic style *exclusively* for the Profession.

| | | | |
|---|---|---|---|
| Block Work, carved and fitted at | 50 cents | per | Tooth. |
| " " and mounted | $1 25 | " | " |
| Continuous Gum Work - - | 1 25 | " | " |
| Single Gum Teeth mounted for | 1 25 | " | " |
| " Plain - - - - - | 1 00 | " | " |
| Gutta Percha Work - - - | 75 | " | " |

Every dentist must furnish his own materials, or if desired, I will furnish at the regular prices. The above are the prices for full arches, or nearly so. Cases of less than six teeth will be charged 25 cents per tooth, extra.

As we *purchase* and pay for all our teeth, they are subject to our own discretion, without the control or interference of any person. We are, therefore, prepared to offer *inducements* which can not be equalled anywhere else. Those who purchase by the hundred, or use many teeth, will do well to investigate the matter.

### Promiscuous Teeth,

From which to select for odd cases, is important for every Dentist to have on hand, ready at a moment's warning, to select what is needed for a case. The loss of time, expense of postage, disappointment and annoyance of sending to a Depot for one or two teeth, is generally more than the value of the teeth four times over. It is a false economy to have no teeth at all on hand. Every Dentist should have a supply of several hundred for odd jobs.

We have on hand an accumulation of promiscuous teeth, consisting of central and lateral incisors and cuspidatis, which we are disposed to sell cheap by the hundred, viz:

Gum Teeth, $12 per hundred.
Plain " 6 "

We look upon this as a mutual accommodation, and are, therefore, willing to make a sacrifice far below the original cost. At the same time, we are ever ready to select to the best of our ability, to casts, patterns or directions, anything that is wanted, from a single tooth to a full set, or any quantity desired.

### Orum & Armstrong's Teeth.

After a careful observation of years, both personal and by conversation and communication with many of the most prominent Dentists in the profession, we feel called upon, in duty, to notice these teeth as we are convinced they deserve.

The first productions were in some respects faulty—the most beautiful wanted strength, and the strong ones fell short in beauty; but now every obstacle is overcome, and they stand forth prominently—their *beauty attracts* every eye, and their *strength astonishes* every one who tries them.

With a beautiful translucent body, giving the point the natural transparency of the enamel—with the body so handsomely colored and the colors so nicely blended, as to give the full expression of the natural *bone*, these teeth fill a vacuum long felt by the profession. Their natural life-like expression and shapes would, with a very little stretch of the imagination, lead an observer to fancy he saw the blood circulating through the small arteries, giving it brilliancy and vitality.

Great improvements have also been made in the moulds, with fine, healthy gums, and the body and gum so shaped as to require but *very little grinding* to fit them to the plate.

A full set, well mounted and in the mouth, will defy the keenest criticism of any but a skillful and practised eye to tell whether they were placed there by the hand of nature or of art.

"They hold the *mirror* up to *nature*."

It is not difficult to make a *handsome* too h, neither does it require much skill to make a *strong* tooth ; but the great desideratum is to combine in the highest degree STRENGTH and BEAUTY, and here we have it in *perfection!*

Let those who, through prejudice or other feelings, have stood aloof, give them one fair trial, and they will be convinced of their great merits. The little time and labor required in grinding are objects of no small importance to those who have a *large practice*. There are some Dentists who, because they got some of these gentlemen's teeth years ago, and when they were comparatively beginners, and the teeth broke—these gentlemen retain a prejudice which prevents them from using them. Give them impartial trial is all that is desired. The improvement is astonishing, and everything is reduced to a perfectly scientific system, so that at any time and under any circumstances, they can produce and reproduce the same results.

These Teeth are for sale, wholesale and retail, at the Cincinnati Dental Depot, by JNO. T. TOLAND,
38 *West Fourth st., Cincinnati.*

---

### Currency.

The failure and suspension of some of the oldest, and what were deemed the best established and safest banks in the country, has destroyed the public confidence in all bank paper, of which brokers and other money dealers have taken advantage to discredit nearly all the banking institutions in the country, from which they are reaping a rich harvest by purchasing the discredited money, at discounts of 10, 15, 20, 25, and sometimes even greater per centage, and selling gold, silver and eastern exchange at a high premium; and we, with the rest of the commercial community are compelled to stand the shave or let our notes go to protest and be ourselves discredited.

We are frequently told that it is the fault of the merchants themselves—why do they not by common consent receive and pay out the money which they know to be good at par, regardless of brokers? This might be done *in time*, by united and systematic action, but can not be effected in times like these, because the obligations of merchants are already out, contracts made, and all, or nearly so payable in bank, for which we must furnish *bankable funds.*

It becomes, therefore, important that we should have an understanding with our customers, to avoid disappointments, delays, mutual loss, and unpleasant feelings. As an illustration, we will say a gentleman in Illinois sends us $10, with an order for Gold Foil. The money is good where he resides, and probably he has gone to some trouble to get it—as being the best in the place—to make sure of it being all right. We receive it, send it to a broker's office, and find that we can not dispose of it for less than 10 or 15 per cent. discount. Here is a clear deduction of $1 or $1.50; while our profit on the Foil is probably 50 cts., if we take the money at its face, we suffer a clear loss of 50 cts. or $1 below the first cost of the Gold Foil; in fact, the money is worth to us only $8.50 or $9 instead of $10, for which it was sent.

Now, what is to be done? These Goods can be sold *only* for *cash;* the profits are too small to sell in any other way. What's to be done? Are we to send the money back, and let our customer do without Foil until he can send other money in its place, or shall we sell it at the highest price we can get, and send him the value of the *proceeds?* In either case, there is dissatisfaction, unless there is a fair understanding on the subject, and this is what we want to get at.

We have concluded, then, to inform our customers thus publicly what kind of funds are par, and to notify them that where uncurrent money is sent without comment or instructions, to conclude that they are aware of it being so, and accordingly dispose of it to the best advantage, and send them Goods for the amount of net proceeds in par funds.

If the money is at a premium, give them credit for the advance, and if at a discount, charge the loss.

Just at this time exchange on New York and gold and silver command a premium of from 5 to 10 per cent.; to-morrow it may be higher or lower. The only Bank paper which is taken at par in this city are as follows:

State Bank of Ohio,
State Bank and Bank of the State of Indiana,
Kentucky, except Ashland,
Louisiana,

We have no assurance that these will continue solvent, altho' we have strong faith and hope. If they should fail or suspend, they must, of course, go with the other depreciated currency.

Gold and silver are really the only *certain* par funds ; exchange on New York is worth as much premium, provided the draft is drawn on a solvent house, one that has not or will not fail before the draft reaches its destination.

---

### Jones, White & M'Curdy's Teeth.

These are of such wide-spread reputation, and so well known by the profession, that we deem it unnecessary to comment on their beauties or merits. Nearly every Dentist in the United States has used them more or less, and can, therefore, better judge how they suit him than we. We have, therefore, only to inform our friends and the profession that we have on hand a large stock, carefully selected, of all shades and sizes, of the latest patterns, in full sets, sets of six, and promiscuous, which we are prepared to sell, *wholesale* or *retail*, at the *lowest market* prices.

JNO. T. TOLAND,
38 *West Fourth st.*

---

### John Klein's Porcelain Teeth.

These teeth are offered to the profession by the manufacturer, with confidence that they will give satisfaction. There are some of the shapes and shades very good, and not to be found in any other stock. An extra inducement is offered in the prices :

| | |
|---|---|
| Plain Plate Teeth (front 6) - - | 8 cents. |
| " " " (Molars and Bicuspids) - | 9 " |
| Gum Teeth, - - - - | 18 " |

JNO. T. TOLAND, Western Agent,
38 *West Fourth st., Cincinnati.*

---

### India Rubber Work.

Considerable inquiry having been made regarding India Rubber work, we would state for the information of those who wish to experiment or engage in this work, that the process is held under " Goodyear's Patent." Mr. Goodyear is at present in Europe, and has made no arrangements for the disposal of rights.

We can furnish the necessary apparatus for attaching to other steam works for $35, and all necessary materials for doing the work, at the market price. Purchases in this line must in all cases be strictly cash, as we have to advance the money for all materials and apparatus in this work.

When Mr. Goodyear returns, he will make arrangements for the disposal of office and other rights, consequently, any purchases made are subject to his claims, either to purchase a right or discontinue the work.

---

### Lawson's Patent Furnace and Blowpipe.

The whole combination of this blowpipe is so complete and simple that it perfects this part of the dental profession.

The combination of the safety valve B, guage cock D, and lamp M is so peculiarly arranged that any blast required by the dentist can be had in an instant by changing them as directed in the bill accompanying the instrument. By the guage cock D, the blast can be changed from one pipe to the other in one second of time, and changed back in the same time.

With this instrument any one can braze a fine watch hand or a solid metallic substance one inch in diameter, and in short, everything in the shape of a heat required by the Dentist, can be done with this instrument. Price $12.00.

Of these we have a fine stock on hand. They are undoubtedly the best article of the kind ever offered.

—ALSO—

### Self-acting Blowpipes, of various kinds.

| | |
|---|---|
| Tin Frame, - - - - | $3.50 |
| Copper Boiler, - - - - - | 4.50 |
| Brass " and Stand, - - - - | 5.00 |

---

### Chairs, Cases, and Rolling Mills.

A good assortment of all the most approved kinds, at the lowest prices.

### Dentists' Forges.

We keep on hand a supply of Queene's celebrated Forge, which are neat, convenient, and cleanly, requiring very little fuel (charcoal), and in summer throws out no heat.

Dentists' size, with pot and slides, - - $27.00

—ALSO—

### Barron's Blast Furnace.

This is a neat and convenient Forge, suitable for dentists and jewellers, producing an intense heat in a few minutes, and at very low price. Price, with bellows and one joint of pipe, $10.

---

### Furnaces,

FOR MAKING CONTINUOUS GUM WORK.

I keep on hand at all times a supply of the most approved kinds, at the lowest prices.

---

### Correction.

In our Catalogue, recently issued, in describing and recommending Cases for Instruments, we gave a special description of what is called a Five Drawer Case, but neglected to state that the profession are indebted for the invention of this haudsome and convenient arrangement to Mr. J. D. Chevalier, of New York City. This beautiful style of case is by many known as the Chevalier Case.

I am just in receipt of some of these cases, and have ordered several more of the most approved and convenient sizes, which I will be pleased to furnish to Dentists, either empty or filled with handsome and useful instruments, at the lowest Eastern prices.

We pride ourselves somewhat on the arrangement and style of cases fitted up by us, and would invite all who wish to procure an outfit, to call and examine before purchasing, believing that we can convince the most skeptical of the superiority of style and workmanship, as well as economy in price, over all others.

### Sherwood's Forceps.

These are manufactured by Mr. H. R. Sherwood *exclusively for my sales*, according to the most approved patterns, *perfectly* adapted to the *hand* and *tooth*, enabling the Dentist to operate with *ease* and *certainty*.

☞ N. B. We regret to state in this connection that there are dealers who deem it to *their interests* to endeavor to bring our instruments into disrepute, by having the same shaped instruments made by *inferior workmen*, and then advertising " Sherwood's Patterns " at prices far below our prices, and as much below what they sold them at whilst *they had them* for sale.

We will, however, trust to the *honor* and GOOD SENSE of the Profession (which thus far has not failed) to discriminate between the GENUINE article, which has received the sanction of their universal approval, and an IMITATION of (to say the least) *doubtful* quality.

☞ Whenever a Dutchman ("over the Rhine") can be found to make Forceps EQUAL in any respect to those made by Mr. Sherwood, we will then think of entering into competition, but until then, those willing to be deceived, are welcome to their BARGAINS.

The following is a list of our regular Shapes, of which we endeavor to keep a constant supply on hand, although at present we are over-crowded with *orders*. The prices are the same throughout, regardless of shape. Any other patterns will be made at the same prices, as follows :

| | |
|---|---|
| Broad Fancy Handles, octagon joints & jewelled, shell pattern, each, - - - - - | $4 00 |
| Broad Fancy Handles, octagon joints & jewelled, fish pattern, each, - - - - | 3 50 |
| Broad Fancy Handles, oct. joints, without jewelled, each, | 3 00 |
| Broad plain " " " Jeweled, " | 3 00 |
| " " " " " without " " | 2 25 |
| Narrow " " " " like Eastern " | 2 00 |
| Broad Oval jointed Forceps, first quality, " | 2 00 |
| Narrow " " " like Eastern, " | 1 75 |
| " " " " 2d quality, " | 1 50 |
| Forceps for the Superior Dentes Sapientia. | |
| " " " " " Roots. | |
| " " Inferior " " | |
| " " " " " Roots. | |
| " " Elevating the roots of " | |

Forceps for the Superior Molars, Right Side.
" " " " Left "
" " " " " " Roots.
" " " " Right " "
" " Inferior " " "
" " " " Left "
" " " " Either "
" " " " Roots.
" " Superior Bicuspids, Either Side.
" " Inferior " Right "
" " " " Left "
" " " " Either "
" " Superior Incisors, " "
" " Inferior " " "
" " Superior Roots.
" " Inferior "
" Narrow Beak Hawk's Bill for crowded Teeth.
" for Separating Roots of Teeth.
" " Excising Teeth Straight.
" " " " Curved.
Light Forceps for Children's Teeth at corresponding prices.
Prof. Harris' patterns of Forceps.
" Taylor's " " "
Dr. W. M. Hunter's patterns of Forceps.

| | |
|---|---|
| Dr. Elliot's Pivot Extractor, - - - | $3 00 |
| " Hullihen's Screw Forceps, - - | 2 50 |
| " " " " with Chevalier's Improvement | 3 00 |
| " Baxter's Plugging Forceps, plain, - - | 5 00 |
| " " " " Pearl sides, - | 8 00 |

---

### Forceps for Physicians.

Shaped to the tooth and hand, made of good metal and neatly finished.

Upper Molar, with curve or hook for little finger, either side.
" Bicuspid " " " "
" Incisor, Straight.
Lower Molar and Dentes Sapientia, Either Side.
Lower Fang, curved.
Upper Fang, straight.

Price of each, $1 25.

—ALSO—

Common Forceps, consisting of straight and curved.

| | | |
|---|---|---|
| Single jointed, | - - - - | 60 cents each. |
| Double " small, | - - | 75 " " |
| " " large, | - - - | $1 00 each. |

A liberal discount to Dealers who purchase by the dozen to sell again.

---

### Chevalier's Forceps and Instruments.

I am prepared to supply the profession with every kind and description of Instruments, manufactured by Mr. J. D. Chevalier, for the same prices at which they are sold at his establishment in New York.

*Also,*

H. G. Kern's instruments, at his prices.
J. H. Gemrig's instruments, "
And all others at the respective manufacturers' prices.

A full and complete Catalogue of Goods and prices will be sent by mail to any part of the country on application.

---

### Remittances.

Just at this time this is a subject of much importance as to kinds, etc. As a general rule, Drafts on any of the Eastern cities are at a premium, especially on *New York*, whilst Drafts on any point west, south or north of us are at a *discount*. If, therefore, you wish to make a remittance by draft to Cincinnati, purchase your draft on New York; you pay a premium of, say 5 per cent.—send it here, and we sell it for 4 or 5 per cent., and give you credit for the amount of the draft and premium, and you lose nothing, because you get the premium back. If you send a draft on any Western city, we have to sell it at a *discount*, and you not only lose the premium paid, but also the discount here. A case in point: A gentleman visiting the city a few days ago, brought with him a draft on Chicago, for which he paid 3 per cent. When he arrived here, the best he could do was to sell at 8 per cent. dis., and he lost 11 per cent. on the amount; he could have bought a draft on New York for 5 per cent., and could also have sold it here for 5 per cent., and thus he would have lost nothing.

All remittances must be in par funds, or if otherwise, must be reduced to that standard. If money is below par, it is worth just that much less, and for us to take it at par, is equivalent to selling the goods at so much less. Very few of our goods will stand it. Gold, platina and silver we sell at from 4 to 6 perct. How, then, can we take money which is 10 per cent. discount? It is a losing business to us, and it were better for us to keep the Goods.

The quotations of "Bank Note Reporters" are at present no guide at all. Illinois money is quoted at from 1 to 2 per cent. discount, yet it is impossible to dispose of it at less than 3 or 10 per cent. The only funds that can be relied on now is State Bank of Ohio, State Bank of Indiana, Bank of the State of Indiana, Kentucky, and Gold and Silver. It is folly to send any other kind, unless you have some kind on hand, and desire to dispose of it at whatever it is worth in the market.

We give below the present rate of discount on currency:

Ohio State Stock Banks are all par, except Springfield Bank, at Springfield, and Miami Valley Bank, Dayton. These are 10 per cent. discount.

State Bank of Ohio and Branches, *par*, except Union Bank, Sandusky City, 10 per cent. discount. Independent Banks, par to 10 and 35 per cent. discount.

Kentucky—all solvent banks *par*.

Virginia, 5 to 10 per cent. dis., except Kanawha, 50 cts.; Transalleghana, 35.

Indiana Stock (specie paying), 5 per cent. dis. Bank of the State and State Bank of Indiana, *par*.

Pennsylvania—Philadelphia Banks *par*, except Bank of Pennsylvania, 50 per cent. dis. Penn. Country Banks, from 2 to 10 per cent. dis.

| | | | | |
|---|---|---|---|---|
| Missouri, | all solvent Banks, | | | par. |
| Illinois, | " | " | | 3 to 10 per ct. dis |
| Wisconsin, | " | " | | 5 to 10 " " |
| Michigan, | " | " | | 5 to 10 " " |

Tennessee. The majority of the Banks in Tennessee having failed, there are no regular rates. Those that are taken at all are 15 per cent. discount.

| | |
|---|---|
| Louisiana, - - - | par. |
| North Carolina, - - | 10 per cent. dis. |
| South Carolina, - - | 10 to 20 dis. |
| Georgia, - - - | 10 to 50 " |
| Alabama, - - - | 15 " |

Maryland—Baltimore city, 5 per cent. dis. Country Banks, 10 to 50.

Delaware, - - - - - 5 to 25.

New Jersey—A few of these are par; the majority are 3 per cent dis.; the remainder are 25 to 50.

New York—N. Y. City, par. Country Banks mostly 3 per cent discount. A few are par, and some from 25 to 75 per cent. discount.

Massachusetts (Boston) par, 1½ and 5 per cent. dis. Country Banks, 3 to 4 per cent. dis.

Rhode Island, mostly 10 per cent. A few are from 15 to 75 per cent.

Connecticut, mostly about 3 per cent. dis. A few are from 25 to 75 per cent.discount.

| | |
|---|---|
| New Hampshire, - - - | 3½ per ct. dis. |
| Maine, - - - - | 3½ " " |
| Vermont, - - - - | 3½ " " |
| Canada, - - - - | 10 " " |

The above is not given as a standard, for we can not anticipate the changes; before this reaches you, the rates may have changed, and may be twice as high, or only half as much; but whatever it may be, we give you all we can get for it, and being acquainted with the Brokers, we can generally do a little better than the published rates.

I have given more space and attention to this subject than intended, but it seemed to demand. Scarcely a day passes that we do not receive uncurrent money, 5, 10, 15, 20 or 25 per ct. discount; we can not use any but *par funds.*

☞ Hereafter we will *sell all* the *money* received immediately, and send goods or give credit for the amount of the *net proceeds.*

If there are any who do not wish to dispose of their money at the market rate, they will please state the fact in their letters.

---

### *A New and Convenient Method of Coating Silver or any other of the Inferior Metals with Gold.*

Take the plate and rub on it a thin coat of mercury (quicksilver); then take a leaf of Gold Foil, spread it over the plate, press it down, and hold the plate over the flame of a spirit lamp for a few minutes, then with a burnisher press down the foil and burnish. You can thus repeat the coatings of mercury and foil to any desired extent, and thereby coat as thickly as desired, with pure gold, perfectly and inseparably.

*The Cause of the present Crisis given in a Nut-shell.*

The only method of making money scarce in this country is that which we adopt of maki..g debt plenty, by which money is made *relatively* scarce. Two-thirds of our currency is debt—a mad system of kiting between the banks and their customers, and an enormous superstructure of debt is built thereon, keeping almost every trader in danger of bankruptcy. *There is nothing else the matter with the business of the country.—Hunt's Merchants' Magazine.*

---

### Ohio College of Dental Surgery.

We publish on another page, the advertisement of this school (gratuitously), believing the object deserving of such effort. We desire to see the school sustained and prospering, and we like to see the young men of the profession starting out in life with the strength of knowledge, theoretic and experimental, which will guarantee success, and make them an honor to the profession and a benefit to their fellow-men.

We would like to see the lecture-room full this winter. We know the times are hard—you can, therefore, the better spare the *time;* the way to make the *times easy* for each one personally is to qualify himself the more thoroughly to be prepared to meet and surmount every obstacle that may be found to impede your advancement and success. Remember that in all things "KNOWLEDGE IS POWER!" Money spent in acquiring knowledge, like investing in a handsome and substantial outfit, is only lent, and, unlike the bread cast upon the water, will return in a very few days.

---

### Prices, Inducements, etc.

I commenced the business proclaiming that I would sell goods at Eastern prices, all except a *few heavy* goods, to which freight would be added. I have done all I proposed, and more, by selling Lathes, Rolling Mills, etc., just as cheap as they are sold East. On Furnaces and a few other articles I can not do this, because the freight costs me much more than I can get discount from regular prices; and I am now ready to take the *Catalogue* of any *Eastern* House, and supply goods at those prices throughout. In bringing the prices down in this market to the Eastern standard, I did not suppose that any would expect or desire them lower; but in this I find I was mistaken, and I also learn that their desires are gratified by secret discounts, presents, etc. I wish it, therefore, to be distinctly understood that as I purchase mostly for *cash*, my experience enables me to purchase and manufacture at the lowest possible figures, and, therefore, no man can or shall undersell me on the same kinds and qualities of Goods.

Manufacturing nearly all our own instruments and dental furniture, and purchasing others from *first hands*, I wish it to be distinctly understood that unless you have a special preference for dealing at some other house, and are not prejudiced against the house or the place, you can do better here than any other place, for if this is to be a "free fight," you can "*consider me in*." And bear in mind that no inducement in the reduction of *prices* or *discounts* can be offered by any *responsible* House, either East or West, that I am not prepared to *equal* or *excel*. As I have determined to concentrate everything into the business, I am also resolved to make it worthy of the name of *the* DENTAL DEPOT OF THE WEST.

### Ohio College of Dental Surgery.

### SESSION OF 1857–8.

The regular course of Lectures in this institution will commence on the first Monday of November, and close on the third Wednesday of February.

**FACULTY.**

C. B. CHAPMAN, Professor of Anatomy and Physiology.
J. B. SMITH, M. D., Professor of Pathology and Therapeutics.
JAMES TAYLOR, D. D. S., Professor of the Institutes of Dental Science.
J. TAFT, D. D. S., Professor of Operative Dentistry.
JOSEPH RICHARDSON, D. D. S., Professor of Mechanical Dentistry.
GEORGE WATT, D. D. S., Professor of Chemistry & Metallurgy.
HENRY A. SMITH, D. D. S., Demonstrator of Operative and Mechanical Dentistry.

To meet the wants of those engaged in practice, half-course tickets are issued, thus enabling them to make out a full course of graduation without leaving their homes and practice for an entire session at one time.

Students should bring with them such instruments and textbooks as they may have on hand.

Candidates for graduation must have attended two full courses of Lectures, the last in this school, and must have enjoyed the advantages of a satisfactory Dental pupilage. Certificates of four years' Dental practice, or of one full course in any respectable Dental or regular Medical College, will be received as equivalent to the first course.

TERMS OF ADMISSION.

The tickets are considered as cash, and when not paid for in advance, a note to the Dean of the Faculty, with approved security, will be required.

| | |
|---|---|
| Tickets for the entire course, - - | $100 |
| Matriculation Ticket, - - - | 5 |
| Diploma Fee, - - - - | 25 |

Good and respectable board can be had at from $2.50 to $4 per week.

GEO. WATT, *Dean.*

### Local Anasthesia.

The proprietor, finding that the expense of manufacturing this instrument was such as to leave him no profit at the prices at which they have been advertised and sold, has advanced the price to $15. The price, therefore, will hereafter be $15 for a set of instruments, with full instructions and an office right to use. Cheap enough, in all conscience, if the invention is good—if it is not good, it is worth nothing. They have now been long enough before the profession and for sale, for every one to be able to judge for themselves.

I am frequently asked for my *opinion* in regard to the "Local Anasthesia." Never having witnessed an application of the instrument, I can give only an opinion, and that founded on the experience and opinions of others, based on physiological facts.

In all cases where the nerve is not exposed, and little or no periostial inflammation, and also in all cases where the tooth is dead and the nerve gone, as in clearing the mouth of old stumps and roots, preparatory for artificial work, the instrument can be used successfully and with great advantage; but where the nerve is exposed, or much inflammation exists, the pain of application is as great, or nearly so, as the pain of extraction would be in the ordinary way. The invention can be used even in these cases, but the temperature must be reduced gradually before applying the instruments, and this would frequently cost more time than the Dentist could spare or the patient would be willing to pay for.

There are many cases in the practice of every Dentist, in which a single application would compensate for the entire cost. Instance—A lady, highly nervous and sensitive, afraid to submit to an operation without the use of some anasthetic agent—perhaps in the peculiar state of delicate health in which it would be very dangerous to give much of a shock to the system, or to administer chloroform—one or more teeth or roots are to be taken out and a full set put in, what's to be done? Shall you gratify your patient, and secure a job worth $150, and retain the influence of your patient, or will you let the patient go to some one else, who will use the invention, and thus you lose the work and the influence together?

The remark has been made by several who have used the instrument, that although there occur many cases in which this agent can not be used with advantage, yet for those in which it can be used, they would not do without it for any consideration. Price $15.

---

### "Cheoplasty."

On this subject I think it but just to make some remarks, as my position seems to be misunderstood.

I am neither for nor against it, believing that the Dentist who has a fair opportunity to make practical tests, is better able to judge of its utility than I.

But having had almost daily inquiry by letter and otherwise, relative to the work and materials, and having written to Dr. Blandy, and also to Mr. Wheelright, without receiving a reply, I concluded to investigate the matter for myself, and finding on what I deemed good authority, that neither the teeth, the metal nor the process had the merit of *originality*, whilst at the same time it was puffed to the skies, holding the power and charm of a patent over it, demanding $100 for an office right before any practical information would be given, and knowing that many of my friends, in order to have every improvement as early as possible, would pay the amount, and probably regret it afterwards, I concluded to make a compound metal, possessing the requisites of strength, stiffness, and fusibility desired—to mould smooth and sharp—with a less number of constituents, and of course less corrosive. I also printed directions (not minute), but sufficiently clear for one of ordinary skill in mechanical Dentistry to experiment, and satisfy himself of the merits or demerits of the work, before spending his money for a patent right.

All this is done not as a source of profit, because the printing and postage cost more than I expected to realize from it; but for the purpose of giving my friends and customers an opportunity to try the new work. After experimenting, if they think favorably of the work, and wish to continue it, they can do so, and if they think the patent *valuable* and *valid*, and can pay for a right, they are entirely welcome to do so—in fact, if this or any other work has the merit of an original invention, and is held by a true and valid *patent*, the patentee or owner should expect and receive reasonable compensation.

I do not now, and never have stood in a position of hostility to Dr. Blandy or the patent, but simply as a PROTECTOR of my friends and customers; and I esteem all my customers as friends. Having thus performed my duty, I leave the subject.

I continue to manufacture a metal for casting plates, and sell the same as heretofore, for experiment or use.

Ingots of about ¼ lb. troy, - - - $1 00

1 lb. or more at $3 per pound.

Printed instructions free of charge.

I also hold myself in readiness to give any further information (in my power) that may be desired on this or any other subject relating to Dentistry.

I have also a supply of Oliver & Harrison's Patent Teeth, admirably suited for Cheoplasty or casting; either on cast plates and for gold and silver plates swaged in the usual way, and the teeth held on by casting. Price for Gum Teeth, 20 cts.; plain, 10 cts. Also, Oliver's pamphlet of instructions for all kinds of casting dentures, and repairing the same. Price $1.

---

### Dental Register of the West.

This highly interesting and ably conducted journal has now reached its eleventh volume, growing each year in interest and usefulness. It is devoted exclusively to the Dental profession, and is, therefore, entitled to their special consideration.

It is neat in typography, printed on good paper, and contains more valuable original matter and information than any other Dental Journal in the United States, and is issued regularly and always up to time. Drs. Taft and Watt, the able and gentlemanly Editors, are deserving of great praise for their active energy and untiring zeal in keeping up a publication that does credit to the West and honor to the profession. It can, without fear of contradiction, be pronounced the best and cheapest Dental periodical in the *world*. Price $3 per annum, *in advance*.

---

### American Dental Convention.

The third annual session of the American Dental Convention was held at the Meionaon, in the City of Boston, on Tuesday, Wednesday, Thursday, and Friday, August 4th, 5th, 6th, and 7th, 1857, commencing on Tuesday, the 4th, at 12 o'clock, M.

The members present represented the different States as follows:

| | | | |
|---|---|---|---|
| Maine | 11 | New Hampshire | 19 |
| Massachusetts | 63 | Vermont | 4 |
| Connecticut | 6 | Rhode Island | 6 |
| New York | 32 | New Jersey | 5 |
| Pennsylvania | 10 | Delaware | 1 |
| Maryland | 7 | Virginia | 2 |
| South Carolina | 1 | Georgia | 1 |
| Missouri | 3 | Mississippi | 1 |
| Louisiana | 1 | Tennessee | 1 |
| Kentucky | 1 | Illinois | 2 |
| Michigan | 1 | Wisconsin | 1 |
| Ohio | 6 | | |

Prof. C. A. Harris called the Convention to order.

A letter from Dr. Clark, of New Orleans, Chairman of Business Committee, was read, excusing his absence.

The old committee, a majority of the members of which were found to be present, were requested to prepare business, leaving them the appointment of their own Chairman.

The Minutes were read and approved.

An inquiry was made as to the face of the record in relation to an instrument for producing Local Anasthesia. Due credit was given to Dr. I. B. Branch, of Galena, Ill., as the inventor.

A letter from Dr. Weiber, of Paris, was read, which referred to some Gold Foil which accompanied the letter. A similar article having been produced and discarded long ago by the profession here, after considerable discussion, the whole matter was laid on the table.

The Committee reported the order of business as follows:

1st. President's Address.

2d. Election of Officers.

3d. Debate—What are the best means of securing a healthy Denture?

4th. What are the mechanical appliances necessary to secure the same?

5th. On the best manner of treating Alveolar Abscess.

6th. Discussion on Mechanical Dentistry.

7th. Discussion on Filling Teeth.

Speeches to be limited to ten minutes, unless otherwise ordered.

The President apologizing for not having prepared an address, made a few remarks calculated to cultivate harmony and good feeling.

The various subjects were discussed at considerable length and with much apparent interest, eliciting much valuable information, the results of the experience of the several members.

On the third day, according to previous appointment, on invitation of the Dentists of Boston, the Convention adjourned at half-past one, and proceeded in a body to the steamboat, for the purpose of an excursion down the Bay.

The excursion and visit to Nahant is beautifully and characteristically described in a letter from the pen of "John Phœnix," which we presume every body has read!

The President appointed the following gentlemen as the Committee to prepare business for the next session:

Dr. J. Taft, Cincinnati, O.; Chapin A. Harris, Baltimore, Md.; C. W. Spaulding, St. Louis, Mo.; Benjamin Lord, N. Y. City; Elisha Townsend, Philadelphia.

The Convention adjourned at one o'clock on the fourth day, to meet in Cincinnati on the first Tuesday in August, 1858.

We regret that we have not space for a more lengthy report. Those who feel interested (and we doubt not it will well repay the perusal and expense) will find in the September number of the Dental Register of the West a full report, occupying 76 pages of solid matter.

---

### Files.

I have on hand the largest stock and finest assortment of Dentists' Files ever offered in the West. They consist of every variety of shape of French, English, and American manufactures. Froid's Separating are superior, and taking the place of Stubs'. Gavage's Hf. Rd. are excellent, and the American plug and trimming, especially Jack's patterns, are superb.

### Adhesive Foil, by Arthur.

This is a valuable work, giving full instructions illustrating the author's experience in the kinds of instruments and the peculiar manipulations of adhesive foil. Price, 75 cents.

---

### Wooden Mallets.

Having for a long time observed the *need* of an article of this kind for driving up and bending plates to the cast preparatory to swageing, I have had some made, which I think are very good. They are made of well-seasoned apple tree wood, and will soon become soft and bruised, so as not to mar the plate. Price 50 cents.

---

### Cincinnati Depot for Orum & Armstrong's

CELEBRATED PORCELAIN TEETH,

And all articles manufactured and furnished by them.

38 *West Fourth street.*

---

WESTERN DEPOT

FOR

JONES, WHITE & M'CURDY'S

**CELEBRATED PORCELAIN TEETH,**

And all other articles manufactured and sold by them, at their Depots in New York, Philadelphia, and Boston,

*No.* 38 *West Fourth st., Cincinnati, O.*

### No. 38 West Fourth street, between Walnut and Main,

CINCINNATI.

The whole interior of this establishment has been overhauled and re-arranged, in view of making of it an

### EXCLUSIVE DENTAL DEPOT.

Hereafter our whole *time*, *talents*, and *attention* will be devoted to the interests of the

**DENTAL PROFESSION,**

By being always prepared to supply everything that the most fastidious may desire. We claim to manufacture

### THE BEST INSTRUMENTS

**IN THE WORLD!**

and have now on hand the best assortment of *Fine Goods* to be found anywhere. Our object has been, and is to cultivate

### *A FIRST CLASS DENTAL TRADE.*

By dealing in Goods of the Best Quality, and selling at the lowest prices at which the same *quality* can be had anywhere in the United States, I challenge competition in quality of Goods or prices.

We fearlessly assert that we have now on our list of regular customers a larger *proportion* of

**FIRST CLASS DENTISTS**

than can be shown by any other establishment. And I have a "generous confidence" that the profession in the West and South will appreciate and sustain an

ESTABLISHMENT IN THE WEST,

**DEVOTED EXCLUSIVELY TO THEIR INTERESTS.**

### Home Manufacture.

As the demand for FINE GOODS is growing rapidly, and the "old fogy" idea of going East for everything is dying away, as it, according to all reason and business principles, should, there seems to be some encouragement for us to extend our manufacturing, so as to embrace, along with INSTRUMENTS,

—ALSO—

**CASES, CHAIRS, LATHES, SPITTOONS,**

and all other articles which may strictly be called "Dental Furniture." It is a well known fact that there is no place in the Union where *better* and *cheaper Furniture and Machinery* is made than in CINCINNATI; but in order to manufacture good articles cheaply, it must be done on a large scale. The goods are needed in the West—they are used in the West—in fact, a very large proportion of the goods sold in Eastern Depots is sent West, and used and consumed in the *West!*

Now, what reason or sense is there in sending to New York or Philadelphia for a chair, and paying the same price as in Cincinnati, with the addition of $5 or $6 freight, with the additional risk of injury by transportation, or else an additional expense for Insurance and Exchange?

All such articles can be made and sold in *Cincinnati* just as *cheap* as in NEW YORK, if the profession will only send their orders here, so as to justify us in making up two or three dozen at a time, instead of two or three single articles, and here you save *freight*, save *time*, and get the articles fresh and *new*, instead of being scratched, soiled, and bruised, by transporting from a great distance.

Gentlemen! I am going to "try it on," and have a practical demonstration whether it can be done or not.

My *ambition* is to supply the profession with everything they want, of better quality and at cheaper rates than can be done elsewhere.

There is only one way in which this can be done, viz: to have *sufficient demand* to *enable* us to purchase and manufacture in

LARGE QUANTITIES.

JNO. T. TOLAND,

**DENTAL DEPOT,**

38 *West Fourth st., Cincinnati.*

